# Exploring the versatility of sesquiterpene biosynthesis in guava plants: a comparative genome-wide analysis of two cultivars

**DOI:** 10.1038/s41598-023-51007-1

**Published:** 2024-01-05

**Authors:** Drielli Canal, Pedro Henrique Dias dos Santos, Paola de Avelar Carpinetti, Matheus Alves Silva, Miquéias Fernandes, Otávio José Bernardes Brustolini, Adésio Ferreira, Marcia Flores da Silva Ferreira

**Affiliations:** 1 Department of Agronomy, Center for Agricultural Sciences and Engineering, Federal University of Espírito Santo, Alto Universitário, s/n, Guararema, Alegre, ES 29500-000 Brazil; 2 National Scientific Computing Laboratory (LNCC), Av. Getulio Vargas, 333, Quitandinha, Petrópolis, RJ 25651-076 Brazil

**Keywords:** Plant breeding, Secondary metabolism

## Abstract

*Psidium guajava* L., a fruit crop belonging to the Myrtaceae family, is highly valued for its nutritional and medicinal properties. The family exhibits a diverse chemical profile of essential oils and serves as a valuable resource due to its ecological interactions, adaptability, and dispersal capacity. The Myrtaceae family has been extensively studied for its terpenoids. Genetic studies have focused on foliar terpene yield in species from the *Eucalypteae* and *Melaleucaceae* tribes. To understand the evolutionary trends in guava breeding, this study predicted terpene synthase genes (TPS) from different cultivars. Through this analysis, 43 full-length TPS genes were identified, and approximately 77% of them exhibited relative expression in at least one of the five investigated plant tissues (root, leaf, bud, flower, and fruit) of two guava cultivars. We identified intra-species variation in the terpene profile and single nucleotide polymorphisms (SNPs) in twelve TPS genes, resulting in the clustering of 62 genotypes according to their essential oil chemotypes. The high concentration of sesquiterpenes is supported by the higher number of TPS-a genes and their expression. The expansion for TPS sub-families in *P. guajava* occurred after the expansion of other rosids species. Providing insight into the origin of structural diversification and expansion in each clade of the TPS gene family within Myrtaceae. This study can provide insights into the diversity of genes for specialized metabolites such as terpenes, and their regulation, which can lead to a diverse chemotype of essential oil in different tissues and genotypes. This suggests a mode of enzymatic evolution that could lead to high sesquiterpene production, act as a chemical defense and contribute to the adaptive capacity of this species to different habitats.

## Introduction

Guava (*Psidium guajava* L.) is a fleshy fruit native to the tropical and subtropical regions of Mexico, Central, and South America^1^, and is part of the pantropical tribe Myrteae, which includes 51 genera and around 2500 species. The genus *Psidium*, with approximately 50 recognized species, shows remarkable diversity in Brazil's Atlantic Coastal Forest, Cerrado, and Caatinga biomes, which are considered the center of diversity for this genus^2,3^. Due to its remarkable adaptability and dispersion capacity, guava is grown successfully in many countries, even in neglected soils^4,5^.

The fruit is a rich source of fiber, potassium, manganese, copper, folic acid, and vitamins A and C^6^. Besides the nutritional value of guava, the plant also contains a diverse array of terpenes in its foliar essential oils^7,8^. These terpenes possess a wide range of beneficial properties, including antioxidant^9^, anti-stress^10^, anticancer, analgesic, anti-hyperglycemic, anti-inflammatory^11,12^, antidiabetic^13^, anti-aging^14^, antimicrobial^12^, antihypertensive, cardioprotective^15^ and antibacterial effects^16^. These terpenes also play roles mediating interactions with abiotic stressors^17,18^ and the plant’s biotic environment, contributing to its defense against herbivores and pathogens^19^, as well as attracting pollinators, particularly in neotropical species with fleshy berries that serve as a food source^20,21^.

The content and composition of essential oils may vary significantly among different species, which can be influenced by genetic and environmental factors^22–25^. In the case of *P. guajava*, the essential oil exhibits variation in its chemotypes, which is influenced by the genotype^7,26,27^. Understanding the genes associated with essential oil biosynthesis pathways, as part of genetic breeding programs, enables the identification and selection of specific genotypes. This knowledge, when combined with conventional techniques, facilitates the exploitation of essential oil production for industrial, pharmacological, and agronomic purposes^28^.

Terpenoids derive from a basic five-carbon unit, isopentenyl diphosphate (IPP), and its isomer dimethylallyl diphosphate (DMAPP)^29^. Prenyl transferases combine these building blocks into isoprene diphosphates of varying lengths, including geranyl diphosphate (GPP), farnesyl diphosphate (FPP), and geranyl diphosphate (GGPP), which are the main precursors of cyclic and acyclic monoterpenes (C10), sesquiterpenes (C15), and diterpenes (C20), respectively^30^. In the final steps of terpenoid biosynthesis, these precursors are then modified modified through carbocationic reactions which are controlled by a large number of enzymes called terpene synthases (TPS). These enzymes catalyze the cyclization, hydride shifts, or other rearrangement of precursor molecules to generate a multitude of terpenoids from a few common substrates^29,30^.

In plants, within genomic sequences, the enzymes are coded by members of the TPS gene family, divided into seven subfamilies. The subfamilies related to secondary metabolism, TPS-a (sesquiterpene), TPS-b (monoterpene), and TPS-g (acyclic mono-, sesqui-, and diterpenes), which are three angiosperm-specific subfamilies. The subfamily TPS-d is specific to gymnosperms (mono-, sesqui-, diterpenes) and the subfamily TPS-h is specific to the spikemoss *Selaginella moellendorffii* (putative bifunctional diterpenes). Also, the members associated with the primary metabolism, subfamilies TPS-e and -f proposed to be combined into the group of TPS-e/f (kaurene, mono-, sesqui-, and diterpenes), conserved among vascular plants, and TPS-c subfamily (diterpenes) of angiosperm copalyl diphosphate synthases^29^.

The action of terpenoids on the plant-environment interaction is much reported in the Myrtaceae family due to its remarkable amount of compounds and related genes. The genetic basis of foliar terpene yield has been extensively exploited, mainly in the subfamily recognized by dried fruits, such as species of Eucalypteae tribe, including *Eucalyptus grandis*, *E. globulus,* and *Corymbia citriodora*, which contain the most significant number of complete TPS genes reported in eudicotyledons (70, 69, and 89 complete genes, respectively). Terpene synthase genes have also been identified in *Melaleuca alternifolia* and *Leptospermum scoparium*, with 37 and 49 putative TPS genes, respectively^31,32^. The oil profile pattern in foliar terpenes in these species are the monoterpenes α-pinene and 1,8-cineole^23,29,33^.

Mono- and sesquiterpenes are pivotal constituents in the essential oil composition of *Psidium* neotropical species^34^. Only one study have been identified TPS genes in fleshy-fruited Myrtaceae species^24^, with monoterpenes as the major compounds. *Psidium guajava* essential oils were characterized by sesquiterpenes as their most abundant compounds, with the main constituents caryophyllene, aromadendrene skeletons acyclic, among others^27,35,36^. Thus, in this study, we structurally characterize predicted terpene synthase genes (TPS) from *Psidium guajava* to elucidate evolutionary trends for guava breeding. We also investigate the gene expression patterns among the cultivars with different agronomic traits and essential oil chemotypes. The genomic resource will elucidate the molecular mechanisms governing the formation and variation in the essential oils content.

## Materials and methods

### Plant materials and sequencing

The transcriptomic data for leaves, flowers, and fruit from the Indian cultivar Allahabad Safeda were retrieved from a public database (accession PRJNA472130), as detailed in Supplementary Table S2^37^.

Genomic and transcriptomic data were obtained from sequencing two cultivars (Paluma and Cortibel RM) registered in the Brazilian Ministry of Agriculture (Ministério da Agricultura, Pecuária e Abastecimento-MAPA; http://sistemas.agricultura.gov.br/; accession PRJNA1020439).

Transcriptomic data derived from samples from five distinct tissues (flower buds, immature, young, and mature leaves, roots) were collected and grouped to obtain pools in Cortibel RM. For Paluma, samples from four tissues (immature, young, and mature leaves, roots), were utilized. Each pool represents one biological replicate consisting of samples from four independent seedlings. The seedlings used were four months old and were grown in a greenhouse. All tissues were immediately frozen in liquid nitrogen and stored at − 80 °C until RNA extraction (Table S2).

RNA was isolated from 100 to 300 mg of tissues using the CTAB-based method^38^. The isolated RNA was treated with DNase enzyme for removal of contaminant DNA and cleaned up using RNeasy Plant Mini kit (QIAGEN). The quality and quantity of total RNA were calculated through TapeStation System (Agilent) and Qubit (Thermo Fisher Scientific), respectively. Good quality RNA was further subjected to rRNA removal using RiboMinus Plant kit (Thermo Fisher Scientific). The transcriptome of the two Brazilian cultivars was sequenced using the Illumina PE NextSeq 500 platform.

### Terpene synthase genes discovery

The resulting peptides of prediction of *P. guajava* genotypes were searched against the Pfam-A database locally, using HMMER 3.0 (Hidden Markov Model) with the previously identified C terminal (PF03936) and N terminal (PF01397) domains^39^. We also aligned the sequences from a curated database of plant sesquiterpene synthases using BLAST programs (e-value < 1e10−5)^40^.

The presence of the target domains was verified by the Pfam database (http://pfam.xfam.org/search), the Simple Modular Architecture Research Tool database (SMART; http://smart.embl-heidelberg.de/)^41^. The intron/exon structures, organization, and motif representation of putative TPS were determined using the Gene Structure Display Server (GSDS) program^42^. The conserved motifs, sequence logo and graphical representations were generated utilizing WebLogo 2.8.2 with default parameters (http://weblogo.berkeley.edu/)^43^.

### Phylogenetic analysis

Considering the amino acid sequences of terpene synthases in *P. guajava*, we employed a phylogenetic reconstruction methodology including sequences from *Eucalyptus grandis*, *Arabidopsis thaliana*, *Vitis vinifera* (https://phytozome.jgi.doe.gov/)^44^, *Eucalyptus globulus*^23^, *Melaleuca alternifolia*^45^*, Psidium cattelyanum*^24^ and *Corymbia citriodora*^46^. The alignment was performed using the MAFFT v. 7.4 software with the G-INS-1 algorithm^47^ and optimized using trimAI v. 1.4^48^.

After the alignment, the next step was the search for the best amino acid substitution model using the IQ-TREE 2^49^ with the selection procedure ProtTest^50^. The best-fit model was chosen based on the Bayesian Information Criterion (BIC)^51^. The model created a maximum-likelihood phylogenetic tree file (.nwk) employing 10,000 bootstrapped replicates. The resulting file was subsequently imported into iTOL version 5.5.1 for visualization and editing^52^.

The generated tree was divided into TPS genes associated with the primary metabolism process (subfamilies -c, -e, and -f) and those involved in secondary metabolic pathways (subfamilies a, b, g). Additionally, the functionally characterized terpene synthases were also included in the phylogenetic analysis (Supp. File 1).

### Transcriptomic analysis and differential expression profiling

Three replicates from *Psidium guajava* cv. Allahabad Safeda, two replicates from cv. Paluma and two replicates from cv. Cortibel RM were used to obtain the gene expression patterns. The data were submitted to quality control with the TRIMMOMATIC 0.22 software^53^ with parameters -phred33 LEADING:3 TRAILING:3 SLIDINGWINDOW: 4:30 MINLEN:85 and their quality certified with the FQC Dashboard software 1.5.8^54^. The de novo RNA-Seq approach assembled the transcriptome by TRINITY 2.8.4 assembler^55^. The aligner HiSat2^56^ mapped the filtered reads to the to the *Psidium guajava* draft genome assembly. The R/Bioconductor package Rsubread, using the function of featureCounts performed the counting table of the mapped reads for the following statistical analysis^57^. The R/Bioconductor package DESeq2 conducts the differential gene expression (DGE) test^58^. It is also applied to the R/Bioconductor package apeglm to shrink log-fold change^59^.

### DArTseq-based SNP analysis

Purified DNA samples (1 μg for each sample) from 62 Brazilian guava genotypes (cultivated and naturalized) were prepared according to recommendations (https://www.diversityarrays.com/faq/) and sent to Diversity Arrays Technology Pty. Ltd company (Canberra, Australia) to identify regions with polymorphism and produce the library using a high-throughput genotyping-by-sequencing system, DArTseq™, and variant calling analysis. Sequencing was made using the Illumina HiSeq2500 sequencing platform, and sequences were processed using proprietary DArT analytical pipelines^60–62^. Barcode/sample sequences were identified and used in label calling. Low-quality sequences were filtered out, and the identical ones gathered into fastqcall files. These files were processed by DArT PL SNP calling pipelines (DArTsoft-seq), as described by Sansaloni and colleagues^62^.

The generated sequences with SNP content (total of 30,761 SNPs markers > 0.9 call rate) from each of 62 genotypes were aligned against the TPS genes with the blastn software 2.8.0 + (evalue 1e−5) to identify highly polymorphic regions.

The heatmap with markers associated with TPS genes was performed using the R software package^63^ and the packages ‘pheatmap’ and ‘RColorBrewer.’ The analysis used the Euclidean distance, and the UPGMA (Unweighted Pair Group Method using Arithmetic averages) clustering method was employed.

## Results

### Identification and expansion of terpene synthase genes

We identified 120 TPS *loci* in the *P. guajava* genome from Brazil assembly (cultivars Paluma and Cortibel RM assembly). Among these loci, the analysis detected 12 pseudogenes (less than three or more than 16 exons), 22 alternative transcripts, and 43 partial genes with only the C-terminal or N-terminal domain (Table S1). Supplementary Fig. 1A, B displays the partial genes and alternative transcripts’ structure and the phylogenetic tree.

**Figure 1:**
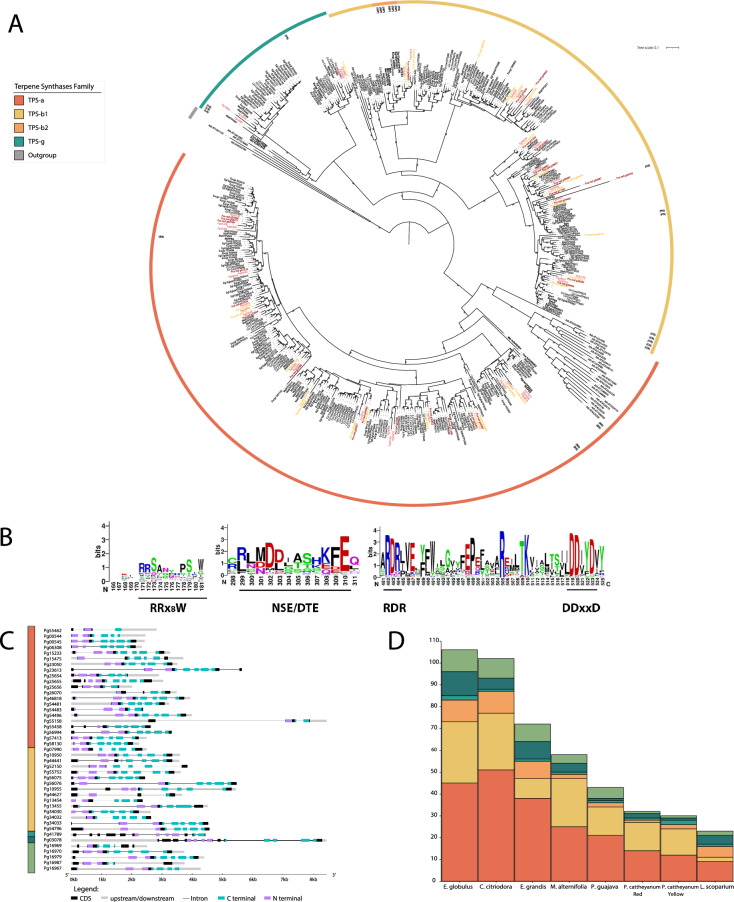
Analysis of distribution, structure, and phylogeny of TPS genes. **(A) **Phylogram based on the Maximum Likelihood Inference of full-length TPS genes. Functionally characterized terpene synthases are written in bold, indicated by the symbols CS (cineol synthase), PS (pinene synthase), BS (caryophyllene synthase), LS (linalool synthase), and ISP (isoprene synthase). Bootstrap support values are indicated near the branch nodes, and values above 80 are displayed. A few genes from *A. thaliana* from TPS-c and TPS-e/f clades were used as the outgroup. **(B)** Conserved motifs representations from all TPS genes, RDR, DDXXD, NSE/DTE and RRX8W, using WebLogo server. **(C)** Gene structure of the 43 putative functional TPS genes from *P. guajava*. Exons are shown as boxes, while introns are shown as lines. The position of the two conserved domains N-terminal and C-terminal are shown in purple and blue, respectively. **(D)** The number of genes in each subfamily relative to the total genes’ number indicates the proportion of TPS genes found in Myrtaceae species.

The phylogenetic analysis used the Maximum-likelihood estimation of forty-three full-length genes of the *P. guajava* genome assembly. We identified five well-supported and very distinct clades (bootstrap = 100) corresponding to the TPS subfamily (i.e., subfamilies a, b, c, e/f, and g), including the JTT + R6 and JTT + F + I + G4 amino acid substitution model, respectively^64^. Specifically, we focused on subfamilies TPS-a, TPS-b, and TPS-g, which are associated with the biosynthesis of secondary metabolites. Our phylogenetic tree incorporated 468 sequences for this category, as depicted in Fig. 1A. In contrast, subfamilies TPS-c, -e, and -f are primarily responsible for producing fundamental metabolites such as gibberellin and abscisic acid. This analysis encompassed a comprehensive set of 44 sequences, featuring representatives from other Myrtaceae species, as illustrated in Supplementary Fig. 1C.

Most genes (21) clustered in a clade corresponding to TPS-a subfamily, recognized for their role in generating sesquiterpenes (C15). Additionally, the TPS-b subfamily was observed, with a predominance of fifteen members classified as TPS-b1, which produce cyclic monoterpenes. Notably, the research identified no TPS-b2 subfamily members responsible for encoding isoprenes and ocimenes (C5, C10). Five subfamily TPS-g members were identified, producing acyclic mono-, sesqui-, and diterpenes (Fig. 1A). One member of the class TPS-c (diterpenes producer) and one member of TPS-e/f (mono-, sesqui-, and diterpenes producer) were identified and participated in the primary metabolic process (Fig. S1C).

Multiple sequence alignment demonstrated that proteins had highly conserved aspartate-rich motifs (DDxxD) and less conserved NSE/DTE motifs at the C-terminal, and an RRx_8_W domain at the N-terminal (Fig. 1B). The aspartate-rich motifs harbor a sequence of 35 amino acids located downstream of the RXR/RDR motif, which serves a crucial function in the chelation of the diphosphate group after substrate ionization. The TPS-c subfamily is present in land plants and is characterized by the “DXDD” motif but not the “DDXXD” motif in their proteins, which was detected in only one guava TPS (Fig. 1B).

About the structure of the twenty-one (49%) TPS-a, most contain 5 to 8 exons, except for Pg55158, Pg54483, Pg26070 and Pg25656, with 3 and 4 exons (Fig. 1C). The TPS-b gene subfamily was the second largest, containing 6 to 9 exons and included 15 genes, about 35% of the total TPS genes, except Pg44627-b1, Pg13454-b2, Pg34032-b1 that contain less than five exons. Also, five genes represented the TPS-g subfamily (which predominantly produces acyclic mono-, sesqui-, and diterpene) and include 6 to 8 exons. For the remaining TPS subgroups, one gene encoding copalyl diphosphate synthase represents the TPS-c subgroup, and one represents the TPS-e/f subgroup. Genes that belong to both TPS-c and TPS-e/f contain 12 and 13 exons, respectively (Fig. 1C).

We also observed predominance of the TPS-a genes transcripts as a proportion of the total TPS genes: 49% compared with *E. grandis*, the next highest at 54%, followed by *V. vinifera* (44%), *C. citriodora*, (45%), *E. globulus* (43%), *M. alternifolia* (43%), and *P. cattleyanum* (40–43%) (Fig. 1D).

In addition to a comparative study of TPS, the study assessed the overall similarity of protein sequences among guava samples compared to other members of the Myrtaceae family. Guava shared only nine complete TPS genes in orthologous pairs (a single gene in one species more closely related to a single gene in a different species than a gene within its genome) with red and yellow araça, then the other rosids. However, it’s worth noting that none of the TPS genes in guava exhibited orthologous relationships with pairs from other eucalyptus species (Table S6). Conversely, in contrast to this observation, eight genes in the yellow morphotype of *P. cattleyanum* were found to occur in orthologous pairs with guava. In comparison, nine genes exhibited orthologous relationships with the red morphotype of *P. cattleyanum* and guava. Interestingly, only nine genes were orthologous with both red and yellow morphotypes of *P. cattleyanum*.

### Global expression profiling of terpene synthase genes from *Psidium guajava*

**Figure 2:**
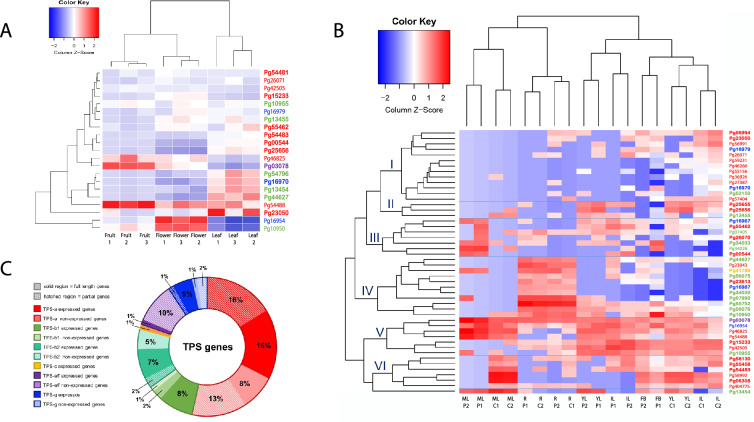
Expression of terpene synthases in various guava tissues. Heat map compares relative transcript abundance for TPS genes in fragments per kilobase of transcript per million mapped reads (FPKM) across the tissues and hierarchical cluster analysis. Each gene (row) is normalized to the percent of total expression for each gene. Red color represents a higher percentage of total expression for a given gene, and blue represents a lower percentage of total expression. Complete genes are highlighted in bold font (contain both domains, PF01397 and PF03936) and subfamilies are in the same colors as the legend of this figure (**C**). The correlation heatmap with a hierarchical cluster shows the tissue matrix (x-axis) and the terpene synthase gene expression matrix (y-axis). (**A)** Heat map shows expression of terpene synthases in three tissues (triplicates) of *Psidium guajava* cv. Allahabad Safeda and hierarchical cluster analysis. (**B)** Heat map and hierarchical cluster analysis of terpenes synthases in four tissues (duplicates) of *Psidium guajava* cv. Paluma, and five tissues (duplicates) of cv. Cortibel RM. *IL* immature leaf, *YL* young leaf, *ML* mature leaf, *R* root, *FB* flower bud. **(C)** Graph shows a summary of TPS gene expression using both RNA-Seq data sets.

To explore the plasticity characteristics of gene transcription, available RNA-seq data sets derived from cv. Allahabad Safeda, Cortibel RM, and Paluma were evaluated (Supp. Table S2). The number of fragments per kilobase of exon per million fragments mapped (FPKM) was used to estimate the relative expression levels of annotated genes (Supp. Tables S3, S4). Figure 2A. displays the heatmap illustrating the relative transcript abundance of 21 TPS genes in leaves, flowers, and fruit tissues in three biological replicates from *P. guajava*, cultivar Allahabad Safeda. The hierarchical cluster analysis shows that the TPS genes were more abundant in the leaf tissue and less in the fruit tissue. Three TPS genes (Pg54488, Pg46825, and Pg03078) were highly expressed in fruit. Two of these genes are members of the TPS-a subfamily and one of the TPS-e subfamily. The more expressed genes in flower tissue were Pg16954, Pg10950, and Pg54488, from subfamilies TPS-g, TPS-b1, and TPS-a, respectively. Although the expression of many TPS genes in leaf tissues, the relative abundance is highlighted only for the Pg23050 gene (putative betacaryophyllene synthase) from TPS-a subfamily.

The relative transcript abundance analysis of tissues from cultivars Paluma and Cortibel RM, also showed a coherent grouping of biological replicates for each tissue (Fig. 2B). The highest rates of relative abundance were observed in the mature leaf tissues, both in Paluma and Cortibel RM. Besides, it is possible to identify clusters of TPS genes abundant in the root tissues. In contrast, younger floral buds, immature leaves, and young leaf tissues have a greater variety of expressed TPS genes.

Cluster I in Fig. 2B was characterized by genes expressed mainly in immature leaf tissue from Cortibel RM genotype, as observed for the Pg56994, Pg23050, Pg56991 genes, all from the TPS-a subfamily. In contrast, this cluster includes less abundant genes, mainly mature leaf and root tissue, both for the Paluma and Cortibel RM genotypes. Cluster II (Pg25655, Pg25656, and Pg13455) has a significant abundance in young leaves. The Pg25655, Pg25656 genes are from the subfamily TPS-a. The genes Pg13455 and Pg13454 cluster close to RtTPS1 (*Rhodomyrtus tomentosa*), in the subfamily TPS-b1 and code for putative pinene synthase.

The genes that form cluster III have a more dispersed expression profile in the tissues, but there is an evident abundance in the green tissues from the Paluma genotype. The genes in this cluster are formed from TPS-a, TPS-g, and TPS-b1 subfamilies. Among these genes are a TPS-g subfamily (Pg16967), a putative (3S, 6E)-nerolidol synthase/(3S)-linalool synthase, and the TPS-a subfamily (Pg55462, Pg00544) which code for putative beta-caryophyllene synthase. The major cluster (IV) is expressed primarily on root tissue from both genotypes, and it is richly formed of genes from different families such as TPS-a, TPS-b2, TPS-c, and TPS-g, but predominantly from the subfamily TPS-b1. Interestingly, the research identified genes in the branch of cineol synthases, emphasizing the genes Pg44627, and Pg55752 (TPS-b1).

Clusters V and VI predominantly consist of genes from the TPS-a subfamily highlighted by the high abundance of transcripts Pg06308, Pg55458, Pg58130, and Pg15233, all putative betacaryophynele synthases. The genes belonging to group VI are predominantly expressed in mature leaf tissue of Cortibel RM, whereas those from group V exhibit higher expression levels in young chlorophyll-containing tissues. Clusters IV and V also have the only members of the expressed TPS-e/f subfamily, Pg03078, a putative linalool synthase, and the TPS-c subfamily Pg41789 encodes a copalyl diphosphate synthase.

Of the total of 86 TPS genes annotated, the expression of 50 (58%) TPS genes was confirmed (Fig. 2C). Among these, 33 (38%) expressed TPS genes containing both domains (PF01397 and PF03936). The RNA-seq experiment of cv. Allahabad Safeda, confirmed the expression of 21 TPS genes. Among these genes, 14 (16%) are complete, and two were expressed exclusively in this genotype (Pg54796-b1 and Pg54481-a). In general, for all cultivars analyzed, there is a greater abundance of genes expressed from the TPS-a subfamily, corresponding to 52% of the total TPS genes expressed in the cv. Allahabad Safeda, and 56% in the Paluma and Cortibel cultivars (Fig. 2C).

### Variations in TPS genes of Brazilian guava germplasm and relationship with leaf terpenic composition

**Figure 3:**
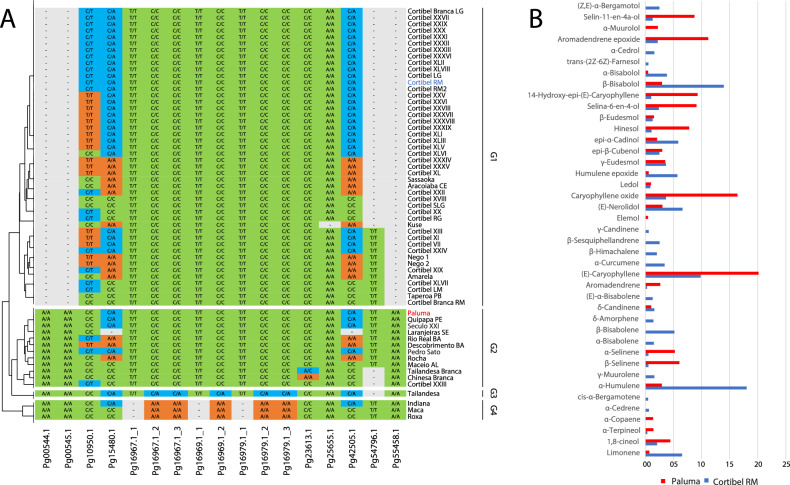
(**A**) Clustering of 62 *Psidium guajava* genotypes based on SNPs in the TPS genes from DArTSeq technology. The two larger groups had genotypes representatives of chemotypes identified for Brazilian germplasm, previously described^7^. The genotypes marked in blue represent the chemotype that mainly presents alpha humulene and beta bisabolol, for example, Cortibel RM. The genotypes marked in red, as Paluma cultivar, represent the chemotype in which most terpenes are B-caryophyllene, and caryophyllene oxide. (**B**) Mean of leaf terpenic compounds identified for the Paluma (orange) and Cortibel RM (blue) cultivars. The values were the means of six independent evaluations of the same genotypes cultivated in different environments and seasons obtained from the studies of^7,26^.

The study analyzed genetic variation within the TPS genes by aligning single nucleotide polymorphisms (SNPs), obtained from genotyping by sequencing using DArTseq, against the guava genome. The study mainly aimed to identify genetic markers for assessing genetic diversity within a comprehensive set of 62 guava genotypes, including Paluma and Cortibel RM varieties, all of which are maintained within a germplasm collection (Fig. 3A). Additionally, this analysis investigated the alternative splicing variants of the TPS genes.

Twelve TPS genes showed SNPs, of which ten were in full-length (five TPSa—Pg00544, Pg00545, Pg23613, Pg25655, and Pg55458; three TPSg—Pg16967, Pg16969, Pg16979; two TPSb Pg54796, Pg10950), and two were partial genes (Pg15480 and Pg42505). Nine of these genes had their expressions detected by RNAseq analysis. The SNPs occurred in the genes’ exons, introns, 3’UTR, and 5’UTR boundaries. Most of them are located in exons (Table S5).

Exploring the SNP positions within the exons, where the triplet of base pairs was responsible for encoding amino acids, data pointed out that the polymorphism occurred in the triplet of Pg15480.1, where the change corresponded to the same amino acid (leucine), indicating a synonymous mutation of the transversion type, as seen in Pg16967.1_2 and Pg16979.1_2 as well. However, the alternative transcripts Pg16967.1_3 and Pg16979.1_3 displayed a non-synonymous transversion mutation, substituting alanine for aspartic acid. Pg16969.1_2 displayed this type of mutation involving the substitution of threonine with asparagine (Table S5). The most variable SNP occurred in the intron portion of the gene Pg10950-b1.

The clustering of the 62 genotypes (Fig. 3A) based on the data of SNPs in the TPS genes reveals two most significant groups, representing the two chemotypes detected in Brazilian cultivars, as exemplified by Paluma and Cortibel RM genotypes^7^. The sesquiterpene prevalence in the essential oils of the mature leaf was also observed in *Psidium guajava* genotypes. Notably, the major compounds identified in the Paluma cultivar included caryophyllene, selinene, cineol, and aromadendrene types. In contrast, the essential oil profile of Cortibel RM was characterized by significant quantities of humulene, trans-nerolidol, D-limonene, bisabolol, α-humulene, and bisabolene (Fig. 3B).

## Discussion

*Psidium guajava*, a native species of America, exhibits remarkable adaptability^5^, broad distribution, and economic, nutritional, and medicinal importance^65^. The essential oils in mature leaves of these plant species show a higher diversity of terpenic compounds, with profiles based on the chemical skeleton^34^.

In the Myrtaceae family, species with dry fruits have the largest numbers of TPS in plants^23,24,45,46^, evidenced by the expansion of the TPS gene and an expansive evolutionary divergence, resulting in often lineage-specific pathways and products^29,66,67^. The contraction of the TPS family in guava, to a certain extent, reflects the loss of redundant genes after whole-genome duplication since the numbers of TPS in species of *Melaleuca alternifolia* and *Psidium cattleyanum* also displayed a slight contraction compared to those found in the eucalyptus lineages^24,45^.

The analyses indicate broad conservation in gene numbers and subfamily representation in the TPS gene family in *Psidium spp*. The red and yellow morphotypes of *P. cattleyanum* share 28–30% orthologous pairs (9 out of 30 and 32; Table S6) and 39% of orthologous pairs with guava (17 out of 43 genes). A comparison of the two eucalypt species (31 out of 52 genes are found in orthologous pairs for *E. grandis* and 31 out of 45 *for E. globulus*) with 59%-68% of genes found in orthologous pairs^23^ shows more conserved genes as the eucalyptus species evolved over a relatively shorter time, approximately 12 million years^23^ than *Psidium* species, which separated about 26 million years of evolution^68,69^. The observed similarities in TPS genes between *E. grandis* and *E. globulus* may be attributed to significant gene family evolution prior to their divergence, and similar mechanisms could potentially operate in *Psidium* species.

The phylogenetic analysis revealed in TPS-a subfamily, five guava genes (Pg23050, Pg46818, Pg54496, Pg15233, Pg57413) closely related to RtTPS3 (AXY92168) from *Rhodomyrtus tomentosa* and EgranTPS038 (Euc_Eucgr_J01451) from *E. grandis*, both belong to a branch of the betacaryophylene synthase, a sesquiterpene^70^. It has been observed that the genes Pg23050 and Pg15233 (full-length) have demonstrated expression within the transcriptome, mainly in juvenile leaf tissues. Consequently, these genes have emerged as potential candidates for functional studies. Two guava genes (Pg00545 and Pg00544) were found in the same branch as RtTPS4 (AXY92169), also putative synthesizing a beta-caryophyllene (Fig. 1A). However, only the gene Pg00544 exhibited expression in the transcriptome, in both mature and immature leaves of the Paluma variety. Although Paluma exhibits higher levels of beta-caryophyllene compared to Cortibel RM and the Allahabad safeda cultivar, the final oil profile may result from the combined expression of diverse genes, contributing in varying amounts to the overall concentration found in the oil.

The prevalence of sesquiterpenes in the essential oils of mature guava leaves was observed in different genotypes. The Paluma cultivar exhibited compounds such as caryophyllene, selinene, cineol, and aromadendrene as major constituents. At the same time, humulene, trans-nerolidol, d-limonene, bisabolol, α-humulene, and bisabolane were the predominant compounds in Cortibel RM^7,26^. The terpenes (*E*)-b-ocimene, caryophyllene oxide, (*E*)-caryophyllene, epi‑a-Muurolol, and epiglobulol were the dominant constituents among the 54 recorded in Allahabad safeda cultivar^72^.

The gene expression across all investigated cultivars revealed a predominant representation of the TPS-a subfamily, constituting 52% of the total expressed TPS genes in the Allahabad Safeda cultivar and 56% in both the Paluma and Cortibel cultivars. This observed expression pattern significantly contributes to the overall composition of essential oil products. Additionally, distinct transcripts were identified in the examined tissues, underscoring the functional adaptability of TPS-a genes. The functional significance of these findings is underscored by the role of sesquiterpenes, identified as crucial signaling molecules in various plant–insect and plant-pathogen interactions^71^. These sesquiterpenes play a pivotal role in attracting pollinators and defending against insect herbivores. Notably, the prevalence of putative TPS-a suggests that sesquiterpenes may have played a key role in driving adaptive traits in *P. guajava*. This is particularly pertinent to the plant's survival and proliferation in the challenging environments of wet forests across the neotropics.

The TPS-b subfamily grouped into two clades. The TPS-b1 clade contains putative cyclic monoterpene synthases. In general, the most observed monoterpenes are cineol, limonene, pinene, and linalool. This study identified 12 genes associated with the expression of monoterpenes in Paluma and Cortibel. Among these genes, some are in the same branch of functionally characterized genes associated with pinene synthase production (Pg13454 and Pg13455), and cineole synthase production (Pg52150, Pg44627, Pg55752), demonstrating their significance as potential candidates for functional studies related to this chemical marker production within the Myrtaceae family^73^.

In maize, the production of β-caryophyllene in the root interacts with the attraction of the root-knot nematode^74,75^. In the context of guava cultivation in Brazil, the root-knot nematode, *Meloidogyne enterolobbi* stands out as a major pest^76^, causing galls and rot, thereby compromising the root system, restricting fruit production and quality, and ultimately leading to plant mortality^77^. Therefore, the study of the TPS-b1 subfamily genes in guava root tissues, especially Pg55752, Pg07990, Pg56076, by their potential involvement in caryophyllene production in root, is fundamental for a deeper understanding of the genetic mechanisms underlying defense responses against herbivores, such as the root-knot nematode, to develop targeted strategies to mitigate the damage caused by this pest in guava cultivation, preserving fruit yield and plant health.

The TPS-b2 subfamily contains putative isoprene/ocimene (C5, C10) synthases (Fig. 1A). Despite ample evidence of the isoprene synthase acyclic genes’ emergence before the emergence of Myrtaceae, no sequences were found in *Psidium* species. This observation suggests that some Myrtaceae could have lost the isoprene/ocimene type of Tps-b2 gene, which arose relatively recently, likely through either whole-genome or localized duplication. A similar loss followed by radiation is apparent in *Arabidopsis thaliana* genes. Isoprene, the smallest terpenoid compound, significantly influences Earth’s atmosphere, enhancing aerosol and ozone formation. In plants, it provides vital functions: heat protection, ozone tolerance, and defense against reactive oxygen species^78^. However, the high biosynthetic cost (at least 20 ATP and 14 NADPH^79^), highlights the ecological significance of isoprene emissions, particularly in high-emitting species like *Eucalyptus*. *Psidium* may not have experienced the same biotic and abiotic adaptive pressures to expand its TPS-b2 subfamily as the eucalypts since they diverged from their most recent common ancestor more than 70 million years ago^80,81^.

Hierarchical cluster analysis showed that TPS genes are more expressed in leaf tissues and less abundant in fruit tissues. Gene expression varied between cultivars, with specific genes from the TPS-a subfamily showing great expression in immature leaf tissues of the Cortibel RM cultivar and an evident abundance in the green tissues from the Paluma genotype with TPS-b1 and TPS-g subfamily. These results demonstrate the functional plasticity of TPS genes in different genotypes and developmental stages, and suggest their significant contribution to the chemical diversity of essential oil compounds in *P. guajava*.

The analyses identified only one copy of the full-length diterpene synthase genes (Pg41789 TPS-c and Pg03078-e/f) in *P. guajava*, originating as a synthase-producing gibberellin precursor (regulatory plant hormone)^29^. The other species from Myrtacaea present more than one copy gene to the TPSe/f subfamily (Supplementary Table S5).

Alternative splicing in the TPS genes was one mechanism of terpenic diversification shown in this work. The mechanism included 24 forms for 17 full-length genes, mostly from TPS-a followed by TPS-b1 subfamilies. The alternative transcripts indicated that the variation in emission might be regulated at the post-transcriptional level, as previously suggested for TPS from the Myrtaceae family^82^. The loss of function in specific terpene synthases due to altered splicing may be one of the causes of intra-specific variability observed in Myrtaceae. The functional significance of this alternative splicing still needs to be directly tested.

The study also focused on investigating variations in TPS genes within Brazilian guava germplasm and their relationship with the terpenic composition of leaves. To analyze the genetic variations, single nucleotide polymorphisms (SNPs) in TPS genes were examined through genotyping by sequencing, including Paluma and Cortibel RM. Twelve TPS genes exhibited SNPs in the population of 62 individuals, with nine showing expressions detected by RNAseq analysis. The hierarchical clustering of genotypes based on these SNPs in TPS genes showed clusters with genotypes representatively of different essential oil chemotypes identified by^7^ as Paluma, Cortibel RM, Cortibel XIII, and Roxa genotypes.

The genomic location of a Single Nucleotide Polymorphism (SNP) can hold functional significance for an individual. An SNP within the coding region can impact protein formation, representing a non-synonymous mutation wherein the base substitution alters an amino acid in the polypeptide chain^83^. Such a mutation was identified in the genes Pg16967, Pg16969, and Pg16979 (Table 1), responsible for (3*S*,6*E*)-nerolidol synthase. This mutation may exert influence by suppressing or favoring enzymatic activity^84,85^.

**Table 1:** Size of typical plant terpene synthase (TPS) family and subfamilies in plant species.

Family	Species	Total TPS	Full length	Full length subfamilies							
				a	b1	b2	c	d	e/f	g	H
Brassicaceae	*Arabidopsis thaliana*	40	33	23	6	0	1	0	2	1	0
Vitaceae	*Vitis vinifera*	152	69	30	16	3	2	0	1	17	0
Salicaceae	*Populus trichocarpa*	57	51	24	14	2	2	0	4	3	0
Funariaceae	*Physcomitrella patens*	4	1	0	0	0	1	0	0	0	0
Pinaceae	*Picea glauca*	83	55	0	0	0	1	53	1	0	0
Selaginellaceae	*Selaginella moellendorfi*	18	14	0	0	0	3	0	3	0	8
	*Melaleuca alternifolia*	79	58	25	22	2	1	0	4	4	0
	*Psidium cattheyanum red*	110	32	14	13	1	1	0	2	1	0
	*Psidium cattleyanum yellow*	106	30	12	12	2	2	0	1	1	0
**Myrtaceae**	***Psidium guajava***	**98**	**43**	**21**	**15**	**0**	**1**	**0**	**1**	**5**	**0**
	*Corymbia citriodora*	127	102	51	26	10	1	0	5	9	0
	*Eucalyptus globulus*	143	106	45	28	10	2	0	11	10	0
	*Eucalyptus grandis*	172	72	38	9	8	1	0	8	8	0
	*Leptospermum scoparium*	49	23	9	2	5	1	0	4	2	0

Significant values are in bold.

The high number of genes, the diversification of subfamily TPS-a, and the more abundance expression in the leaf tissues is consistent with the amount and variability of the terpenic compounds related in the leaf essential oils of *P. guajava*, predominantly of sesquiterpenes^7,26^. As examples of the Paluma e Cortibel RM cultivars studied in this work were related to 42 terpenic compounds, with more than 80% sesquiterpenes, with 17 exclusives of Cortibel RM and three exclusives of Paluma (Fig. 3B), showing genotyping specific profiles of the two chemotypes.

The most common and abundant foliar terpene pattern across Myrtaceae family is α-pinene and 1,8-cineole. We detected its putative genes in guava Pg16970, Pg16987, Pg13454, Pg13455 and Pg55752, Pg44627, Pg52150, Pg44441, respectively. In other species of the Myrtaceae family, such as those belonging to the tribes Myrteae, Kanieae, Syzygieae, Xanthostemoneae, Syncarpieae, and Lindsayomyrteae, these monoterpene are primarily replaced by β-caryophyllene (a sesquiterpene) as the most abundant terpene^24,73^. Caryophyllene has been identified as one of the major components in guava leaf essential oil across different countries^8,34,86–88^, with only a few monoterpenes present in the essential oil composition^34,89^. However, within the *Psidium* genus, for native *Psidium cattleyanum* species from Brazil, 1,8-cineole was the major component found, and for cultivated varieties across the globe, the β-caryophyllene and caryophyllene predominates^34,73,90^. In our study we also detected Pg55458, Pg06308 Pg55462, Pg58130, Pg15233, Pg54496, Pg46818, Pg23050, Pg57413 putative genes to the biosynthesis of β-caryophyllene.

## Conclusions

This study constitutes an extensive examination of TPS genes in guava, involving the thorough identification and analysis of their underlying structural elements. A particular focus is directed towards elucidating the variations within these genes across key cultivars of significance in Brazil. Furthermore, we delve into the evolutionary relationships by examining their connections with other Myrtaceae species. In a culmination of our efforts, we present the expression profiles of TPS across diverse tissues and cultivars, thereby contributing valuable insights into the functional dynamics of these genes in the context of guava.

In summary, the relatively low number of TPS genes between *P. guajava* and *P. cattleyanum* reflects their close phylogenetic relationship. Additional investigations focused on the functional characterization of TPS genes and their regulatory mechanisms shall contribute to a deeper understanding of terpene biosynthesis in guava. During the continuous evolution of guava, copies of some genes were retained, while some losses occurred in other TPS genes.

Analyzing single nucleotide variations in TPS genes in the Brazilian guava cultivars, including alternative splicing forms, reveals significant diversity. This suggests potential implications for gene functionality and the terpenes biosynthesis. The SNPs in functional genes, particularly exonic non-synonymous mutations, could serve as valuable molecular markers for functional gene mapping and genetic improvement of guava for terpene biosynthesis.

The availability of TPS annotation can be valuable for future breeding programs and the selection of guava cultivars with desired terpenic compositions for specific applications in the food, fragrance, and pharmaceutical industries. This research will further enhance our comprehension of the genetic foundations that underlie the observed diversity and adaptability in this economically significant plant species.

### Supplementary Information

Supplementary Figure S1.

Supplementary Tables.

Supplementary Information.

## Data Availability

The sequences used for alignment are presented in the Supplementary Material, available at Scientific Reports’s website. The genome sequence data are available in the NCBI Short Read Archive under BioProject No. PRJNA631442. The transcriptomic data can be obtained through the specified accessions numbers, SRR7186630 and PRJNA1020439. The authors declare that the data supporting the findings are available within the paper.
