# Supplementary material for: Exploring the versatility of sesquiterpene biosynthesis in guava plants: a comparative genome-wide analysis of two cultivars
Source: Sci Rep. 2024 Jan 5;14:574. doi: 10.1038/s41598-023-51007-1 (PMC10770072; doi:10.1038/s41598-023-51007-1)
Supplement: Supplementary file 1 — Supplementary Figure S1. [file 41598_2023_51007_MOESM1_ESM.pdf]

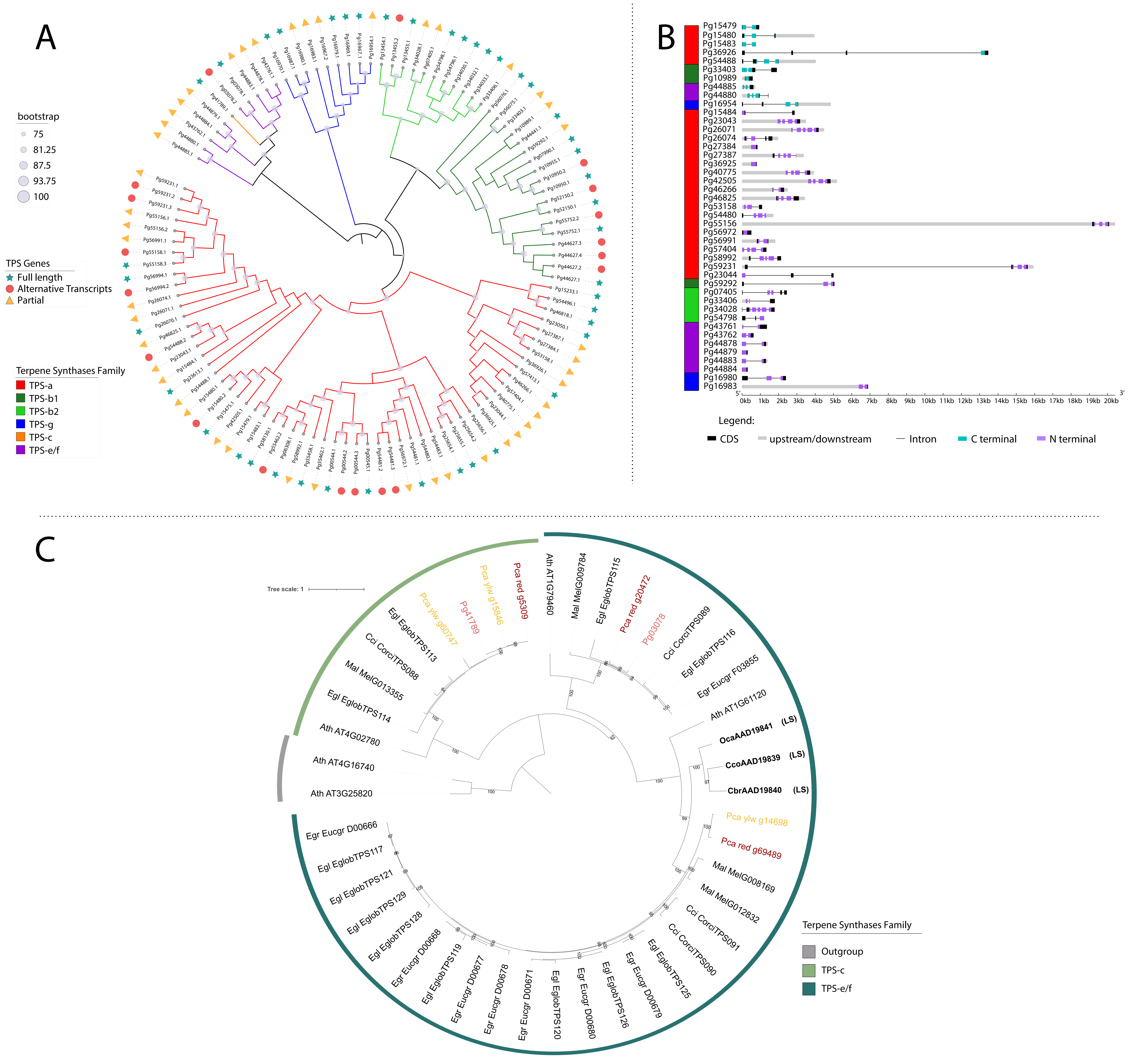

Supplementar Figure S1 – Phylogenetic analysis of terpene synthase (TPS) genes of *P. guajava* including A. full length (blue star), alternative transcripts (red circle) and partial TPS (green triangle). B. Structure of the 43 partial TPS genes. C. Phylogenetic analysis of terpene synthase (TPS) genes involved in primary metabolism across the Myrtaceae family. Functional characterized terpene synthases are written in bold. Bootstrap values support is indicated near the branch nodes and values grater than 80 were shown. A few genes from *A. thaliana* from TPS-b and TPS-g clades were used as the outgroup.
