# Supplementary material for: Exploring the versatility of sesquiterpene biosynthesis in guava plants: a comparative genome-wide analysis of two cultivars
Source: Sci Rep. 2024 Jan 5;14:574. doi: 10.1038/s41598-023-51007-1 (PMC10770072; doi:10.1038/s41598-023-51007-1)
Supplement: Supplementary file 3 — Supplementary Information. [file 41598_2023_51007_MOESM3_ESM.doc]

**Supplementary Material Section**

Supplementary Figure S1 – Phylogenetic analysis of terpene synthase (TPS) genes of *P. guajava* including **A.** full length (blue star), alternative transcripts (red circle) and partial TPS (green triangle). **B.** Structure of the 43 partial TPS genes. **C.** Phylogenetic analysis of terpene synthase (TPS) genes involved in primary metabolism across the Myrtaceae family. Functional characterized terpene synthases are written in bold. Bootstrap values support is indicated near the branch nodes and values grater than 80 were shown. A few genes from *A. thaliana* from TPS-b and TPS-g clades were used as the outgroup.

Table S1. Putative terpene synthase gene in the guava genome and features.

Table S2. The summary of transcriptome sequencing libraries.

TableS3. Transcript abundance for TPS genes in fragments per kilobase of transcript per million mapped reads (FPKM) in three tissues (triplicates) of *Psidium guajava* cv. Allahabad Safeda.

Table S4. Transcript abundance for TPS genes in fragments per kilobase of transcript per million mapped reads (FPKM) in four tissues (duplicates) of *Psidium guajava* cv. Paluma and five tissues (duplicates) of cv. Cortibel RM.

Table S5. Polymorphic Data Within TPS Genes

Supplementary File S1. Functionally characterized genes used in phylogenetic analysis.

| Specie | Acession number | Major product | Reference |
| --- | --- | --- | --- |
| *Abies grandis* | Abi_AAB70907 | limonene | 1 |
|  | Abi_Q9M7C9 | limonene_alphapinene | 1 |
|  | Abi_O24475 | pinene | 1 |
| *Antirrhinum majus* | Ama_AAO41727 | myrcene | 2 |
| *Arabidopsis thaliana* | Ath_P0DI76 | cineole | 3 |
|  | Ath_AAO85539 | betacaryophyllene | 3 |
|  | Ath_AAN65379 | beta_ocimene | 4 |
|  | Ath_AT5G23960 | caryophyllene_humulene | 3 |
|  | Ath_AAU01970 | cineole | 3 |
|  | Ath_NP_189209 | pinene synthase | 5 |
| *Artemisia annua* | Art_AAL79181 | betacaryophyllene | 6 |
| *Artemisia annua* | Art_Q94G53 | betapinene | 7 |
| *Cannabis sativa* | Csa_ABI21837 | limonene | 8 |
|  | Csa_DQ839405 | alphapinene | 8 |
|  | Csa_AAU05952 | betacaryophyllene | 8 |
|  | Csa_A7IZZ2 | alphapinene | 8 |
|  | Csa_ABI21838 | alphapinene | 8 |
| *Camellia sinensis* | CaSi_QID05625 | ocimene | 9 |
| *Citrus junos* | Cju_AAK54279 | betafarnesene | 10 |
| *Citrus limon* | Cli_AAM53944 | limonene | 11 |
| *Citrus unshiu* | Cun_BAD91045 | cineol | 12 |
| *Citrus unshiu* | Cun_BAP75559 | linalool | 13 |
| *Clarkia breweri* | Cbr_AAD19840 | linalool | 14 |
| *Clarkia concinna* | >Cco_AAD19839 | linalool | 14 |
| *Dendrobium officinale* | Dof_DoTPS10 | linalool | 15 |
| *Eucalyptus grandis* | Egr_010046521 | cineol | 16 |
| *Eucalyptus globulus* | Euc_DD464633 | cineol | 17 |
|  | Egl_EglobTPS106 | isoprene | 17 |
| *Eucalyptus polybractea* | EpTPS1_MK873024 | pinene | 18 |
|  | EpTPS2_MK873025 | cineole | 18 |
|  | EpTPS3_QCQ05478 | cineole | 18 |
|  | Epo_MK873026 | cineole | 18 |
|  | Epo_MK873024 | betapinene | 18 |
|  | Epo_MK873025 | cineole | 18 |
| *Fragaria x ananassa* | Fra_CAD57092 | alphapinene | 19 |
|  | Fra_CAD57106 | linalool | 19 |
|  | Fra_CAD57106 | linalool | 19 |
| *Gossypium hirsutum* | Ghi_AGX84977 | alphapinene | 20 |
| *Gossypium hirsutum* | Ghi_AFQ23183 | betacaryophyllene | 21 |
| *Gossypium hirsutum* | Ghi_KJ957818 | linalool | 22 |
| *Hedychium coronarium* | Hed_AGY49283 | linalool | 23 |
| *Lavandula angustifolia* | Lan_DQ263740 | limonene | 24 |
|  | Lan_DQ263741 | linalool | 24 |
|  | Lan_ABB73045 | linalool | 24 |
|  | Lan_JN701461 | cineole | 24 |
|  | Lav_Q2XSC5 | linalool | 24 |
|  | Lav_JN701459 | cineole | 24 |
| *Lavandula latifolia* | Lla_ABD77417 | linalool | 25 |
|  | Lla_DQ421801 | linalool | 25 |
|  | Lla_JN701460 | cineole | 26 |
| *Lavandula pedunculata* | Lpe_AGN72799 | alphapinene | 27 |
| *Malus domestica* | Mdo_AGB14627 | pinene | 27 |
| *Malus x domestica* | Malus_JX848733 | beta-ocimene | 28 |
| *Medicago truncatula* | Mtr_AAV36464 | betacaryophyllene | 29 |
| *Mentha spicata* | Msp_AAC37366 | limonene | 30 |
| *Mentha aquatica* | Maq_AAL99381 | linalool | 31 |
| *Matricaria chamomilla* | Mch_AFM43734 | betacaryophyllene | 32 |
| *Nicotiana suaveolens* | Nic_ABP88782 | cineol | 33 |
| *Oenothera californica* | Oca_AAD19841 | linalool | 14 |
| *Perilla frutescens* | Pse_ACN42009 | linalool | 34 |
| *Picea glauca* | Pgl_ADZ45498 | cineole | 35 |
| *Pinus banksiana* | Pba_JQ240304 | alphapinene | 36 |
| *Pinus taeda* | Pin_AAO61225 | alphapinene | 37 |
|  | Pta_AAO61228 | alphapinene | 37 |
| *Populus trichocarpa* | Ptr_AEI52904 | linalool | 38 |
| *Rhodomyrtus tomentosa* | Rto_AXY92167 | alfa_beta_pinene | 39 |
|  | Rto_AXY92166 | pinene_caryophyllene | 39 |
|  | Rto_AXY92168 | alfa_beta_pinene | 39 |
|  | Rto_AXY92169 | pinene_caryophyllene | 39 |
| *Rosmarinus officinalis* | Rof_EF495245 | pinene | 40 |
| *Salvia officinalis* | Sof_AAC26016 | cineole | 41 |
| *Salvia fruticosa* | Sfr_ABH07677 | cineole | 42 |
| *Salvia rosmarinus* | Sro_ABI20515 | cinenol | 43 |
| *Solanum lycopersicum* | Sly_ADD96698 | betacaryophyllene | 44 |
| *Solanum lycopersicum* | Sly_XP_004231365 | cineol | 45 |
| *Vitis vinifera* | Vvi_001268216 | alphaterpineol | 46 |
|  | Vvi_ADR74221 | beta-caryophyllene | 46 |
|  | Vvi_ADR74204 | beta-ocimene | 46 |
|  | Vvi_ADR74209 | linalool | 46 |

**References**

1. Bohlmann, J., Steele, C. L. & Croteau, R. Monoterpene synthases from grand fir (*Abies grandis*): cDNA isolation, characterization, and functional expression of myrcene synthase, (-)-(4S)- limonene synthase, and (-)-(1S,5S)-pinene synthase. *J. Biol. Chem.* **272**, 21784–21792 (1997).

2. Dudareva, N. *et al.* (E)-β-ocimene and myrcene synthase genes of floral scent biosynthesis in snapdragon: Function and expression of three terpene synthase genes of a new terpene synthase subfamily. *Plant Cell* **15**, 1227–1241 (2003).

3. Chen, F. *et al.* Biosynthesis and emission of terpenoid volatiles from Arabidopsis flowers. *Plant Cell* **15**, 481–494 (2003).

4. Fäldt, J., Arimura, G. I., Gershenzon, J., Takabayashi, J. & Bohlmann, J. Functional identification of AtTPS03 as (E)-β-ocimene synthase: A monoterpene synthase catalyzing jasmonate- and wound-induced volatile formation in *Arabidopsis thaliana*. *Planta* **216**, 745–751 (2003).

5. Theologis, A. *et al.* Sequence and analysis of chromosome 1 of the plant *Arabidopsis thaliana*. *Nature* **408**, 816–820 (2000).

6. Cai, Y. *et al.* A cDNA clone for β-caryophyllene synthase from *Artemisia annua*. *Phytochemistry* **61**, 523–529 (2002).

7. Lu, S. *et al.* Cloning and functional characterization of a β-pinene synthase from *Artemisia annua* that shows a circadian pattern of expression. *Plant Physiol.* **130**, 477–486 (2002).

8. Günnewich, N., Page, J. E., Köllner, T. G., Degenhardt, J. & Kutchan, T. M. Functional Expression and Characterization of Trichome-Specific (-)-Limonene Synthase and (+)-α-Pinene Synthase from *Cannabis sativa*. *Nat. Prod. Commun.* **2**, 1934578X0700200301 (2007).

9. Huang, X. *et al.* Functional characterization of a terpene synthase responsible for (E)-β-ocimene biosynthesis identified in Pyrus betuleafolia transcriptome after herbivory. *Front. Plant Sci.* **13**, 1–11 (2022).

10. Van Schie, C. C. N., Haring, M. A. & Schuurink, R. C. Tomato linalool synthase is induced in trichomes by jasmonic acid. *Plant Mol. Biol.* **64**, 251–263 (2007).

11. Lücker, J. *et al.* Monoterpene biosynthesis in lemon (Citrus limon) cDNA isolation and functional analysis of four monoterpene synthases. *Eur. J. Biochem.* **269**, 3160–3171 (2002).

12. Shimada, T., Endo, T., Fujii, H., Hara, M. & Omura, M. Isolation and characterization of (E)-beta-ocimene and 1,8 cineole synthases in *Citrus unshiu* Marc. *Plant Sci.* **168**, 987–995 (2005).

13. Shimada, T. *et al.* Characterization of three linalool synthase genes from *Citrus* unshiu Marc. and analysis of linalool-mediated resistance against *Xanthomonas citri* subsp. citri and *Penicilium italicum* in citrus leaves and fruits. *Plant Sci.* **229**, 154–166 (2014).

14. Cseke, L., Dudareva, N. & Pichersky, E. Structure and evolution of linalool synthase. *Mol. Biol. Evol.* **15**, 1491–1498 (1998).

15. Yu, Z., Zhao, C., Zhang, G., Teixeira da Silva, J. A. & Duan, J. Genome-wide identification and expression profile of tps gene family in dendrobium officinale and the role of dotps10 in linalool biosynthesis. *Int. J. Mol. Sci.* **21**, 1–22 (2020).

16. Goodger, J. Q. D., Sargent, D., Humphries, J. & Woodrow, I. E. Monoterpene synthases responsible for the terpene profile of anther glands in *Eucalyptus polybractea* R.T. Baker (Myrtaceae). *Tree Physiol.* **41**, 849–864 (2021).

17. Kanagendran, A., Pazouki, L., Bichele, R., Külheim, C. & Niinemets, Ü. Temporal regulation of terpene synthase gene expression in Eucalyptus globulus leaves upon ozone and wounding stresses: relationships with stomatal ozone uptake and emission responses. *Environ. Exp. Bot.* **155**, 552–565 (2018).

18. Kainer, D. *et al.* High marker density GWAS provides novel insights into the genomic architecture of terpene oil yield in *Eucalyptus*. *New Phytol.* **223**, 1489–1504 (2019).

19. Pechous, S. W. & Whitaker, B. D. Cloning and functional expression of an (E,E)-α-farnesene synthase cDNA from peel tissue of apple fruit. *Planta* **219**, 84–94 (2004).

20. Yang, C. Q. *et al.* Isolation and characterization of terpene synthases in cotton (*Gossypium hirsutum*). *Phytochemistry* **96**, 46–56 (2013).

21. Huang, X., Wu, J., Xu, Q. X., Zhang, Y. & Guo, Y. Y. Molecular cloning of the sesquiterpene synthase gene GhTPS1 of Gossypium hirsutum and its induction by the feeding activity of cotton bollworm larvae. *Chin. J. Appl. Entomol.* **49**, 831–838 (2012).

22. Huang, X. Z. *et al.* The terpene synthase gene family in *Gossypium hirsutum* harbors a linalool synthase GhTPS12 implicated in direct defence responses against herbivores. *Plant Cell Environ.* **41**, 261–274 (2018).

23. Yue, Y., Yu, R. & Fan, Y. Characterization of two monoterpene synthases involved in floral scent formation in *Hedychium coronarium*. *Planta* **240**, 745–762 (2014).

24. Landmann, C. *et al.* Cloning and functional characterization of three terpene synthases from lavender (*Lavandula angustifolia*). *Arch. Biochem. Biophys.* **465**, 417–429 (2007).

25. Zhang, T. X. *et al.* Molecular cloning and expression analysis of a monoterpene synthase gene involved in floral scent production in lily (*Lilium* ‘Siberia’). *Russ. J. Plant Physiol.* **64**, 600–607 (2017).

26. Demissie, Z. A. *et al.* Cloning, functional characterization and genomic organization of 1,8-cineole synthases from *Lavandula*. *Plant Mol. Biol.* **79**, 393–411 (2012).

27. Ma, X. *et al.* Characterization of a monoterpene synthase from *Paeonia lactiflora* producing α-pinene as its single product. *Biotechnol. Lett.* **38**, 1213–1219 (2016).

28. Nieuwenhuizen, N. J. *et al.* Functional genomics reveals that a compact terpene synthase gene family can account for terpene volatile production in apple. *Plant Physiol.* **161**, 787–804 (2013).

29. Gomez, S. K. *et al.* Lepidopteran herbivory and oral factors induce transcripts encoding novel terpene synthases in *Medicago truncatula*. *Arch. Insect Biochem. Physiol.* **58**, 114–127 (2005).

30. Colby, S. M., Alonso, W. R., Katahira, E. J., McGarvey, D. J. & Croteau, R. 4S-limonene synthase from the oil glands of spearmint (*Mentha spicata*). cDNA isolation, characterization, and bacterial expression of the catalytically active monoterpene cyclase. *J. Biol. Chem.* **268**, 23016–23024 (1993).

31. Crowell, A. L., Williams, D. C., Davis, E. M., Wildung, M. R. & Croteau, R. Molecular cloning and characterization of a new linalool synthase. *Arch. Biochem. Biophys.* **405**, 112–121 (2002).

32. Irmisch, S. *et al.* The organ-specific expression of terpene synthase genes contributes to the terpene hydrocarbon composition of chamomile essential oils. *BMC Plant Biol.* **12**, (2012).

33. Roeder, S., Hartmann, A. M., Effmert, U. & Piechulla, B. Regulation of simultaneous synthesis of floral scent terpenoids by the 1,8-cineole synthase of *Nicotiana suaveolens*. *Plant Mol. Biol.* **65**, 107–124 (2007).

34. Masumoto, N., Korin, M. & Ito, M. Geraniol and linalool synthases from wild species of perilla. *Phytochemistry* **71**, 1068–1075 (2010).

35. Keeling, C. I. *et al.* Transcriptome mining, functional characterization, and phylogeny of a large terpene synthase gene family in spruce (*Picea* spp.). *BMC Plant Biol.* **11**, (2011).

36. Hall, D. E. *et al.* Transcriptome resources and functional characterization of monoterpene synthases for two host species of the mountain pine beetle, lodgepole pine (*Pinus contorta*) and jack pine (*Pinus banksiana*). *BMC Plant Biol.* **13**, 1–14 (2013).

37. Phillips, M. A., Wildung, M. R., Williams, D. C., Hyatt, D. C. & Croteau, R. cDNA isolation, functional expression, and characterization of (+)-α-pinene synthase and (-)-α-pinene synthase from loblolly pine (*Pinus taeda*): Stereocontrol in pinene biosynthesis. *Arch. Biochem. Biophys.* **411**, 267–276 (2003).

38. Danner, H. *et al.* Four terpene synthases produce major compounds of the gypsy moth feeding-induced volatile blend of *Populus trichocarpa*. *Phytochemistry* **72**, 897–908 (2011).

39. He, S.-M. *et al.* De novo Transcriptome Characterization of *Rhodomyrtus tomentosa* Leaves and Identification of Genes Involved in α/β-Pinene and β-Caryophyllene Biosynthesis. *Front. Plant Sci.* **9**, 1231 (2018).

40. Filipe, A. *et al.* Molecular cloning and functional characterization of a monoterpene synthase isolated from the aromatic wild shrub *Thymus albicans*. *J. Plant Physiol.* **218**, 35–44 (2017).

41. Wise, M. L., Savage, T. J., Katahira, E. & Croteau, R. Monoterpene synthases from common sage (*Salvia officinalis*). cDna isolation, characterization, and functional expression of (+)-sabinene synthase, 1,8-cineole synthase, and (+)-bornyl diphosphate synthase. *J. Biol. Chem.* **273**, 14891–14899 (1998).

42. Kampranis, S. C. *et al.* Rational conversion of substrate and product specificity in a Salvia monoterpene synthase: Structural insights into the evolution of terpene synthase function. *Plant Cell* **19**, 1994–2005 (2007).

43. Mishra, A., Sanchita, Dhawan, S. S. & Sharma, A. In Sight to the Identification and Analysis of Simple Sequence Repeats (SSRs) in Monoterpene Biosynthesizing Plant Species. *Curr. Bioinform.* **11**, 122–130 (2016).

44. Schilmiller, A. L. *et al.* Studies of a biochemical factory: Tomato trichome deep expressed sequence tag sequencing and proteomics. *Plant Physiol.* **153**, 1212–1223 (2010).

45. Li, Y. *et al.* The biochemical and molecular investigation of flower color and scent sheds lights on further genetic modification of ornamental traits in *Clivia miniata.* *Hortic. Res.* **9**, 1–17 (2022).

46. Martin, D. M. *et al.* Functional Annotation, Genome Organization and Phylogeny of the Grapevine (*Vitis vinifera*) Terpene Synthase Gene Family Based on Genome Assembly, FLcDNA Cloning, and Enzyme Assays. *BMC Plant Biol.* **10**, (2010).
